# Supplementary material for: Boulton-Katritzky Rearrangement of 5-Substituted Phenyl-3-[2-(morpholin-1-yl)ethyl]-1,2,4-oxadiazoles as a Synthetic Path to Spiropyrazoline Benzoates and Chloride with Antitubercular Properties
Source: Molecules. 2021 Feb 12;26(4):967. doi: 10.3390/molecules26040967 (PMC7917742; doi:10.3390/molecules26040967)

Electronic Supporting Information for

Boulton–Katritzky rearrangement of 5-substituted phenyl-3-[2-(morpholin-1-yl)ethyl]-1,2,4-oxadiazoles as a synthetic path to spiropyrazoline benzoates and chloride with antitubercular properties

Lyudmila Kayukova[1,*], Anna Vologzhanina[2,*], Kaldybai Praliyev[1], Gulnur Dyusembaeva[1], Gulnur Baitursynova[1], Asem Uzakova[1], Venera Bismilda[3], Lyailya Chingissova[3] and Kydyrmolla Akatan[4]

[1]JSC «A. B. Bekturov Institute of Chemical Sciences», 106 Shokan Ualikhanov St., Almaty 050010, Kazakhstan; e-mail: lkayukova@mail.ru
[2]A. N. Nesmeyanov Institute of Organoelement Compounds, Russian Academy of Sciences, 28 Vavilov St., B-334, Moscow 119991, Russia; e-mail: vologzhanina@mail.ru
[3]National Scientific Center of Phthisiopulmonology of Ministry of Health of the Republic of Kazakhstan, 5 Bekkhozhin, Almaty 050010, Kazakhstan; e-mail:venerabismilda@mail.ru
[4]S. Amanzholov East Kazakhstan State University, 18/1 Amurskaya St., Ust-Kamenogorsk 070002, Kazakhstan.

Table S1. Experimental details and crystallographic information for **5c-e, 6**.

	**5c** $\cdot H_2O$	**5d**	**5e**$\cdot H_2O$	**6**$\cdot H_2O$
Formula	$C_{14}H_{21}N_3O_4$	$C_{14}H_{18}BrN_3O_3$	$C_{14}H_{20}ClN_3O_4$	$C_7H_{16}ClN_3O_2$
Formula weight	295.34	356.22	329.78	209.68
Space group	*P 2₁/c*	*P2₁*	*C2/c*	*P2₁/c*
a (Å)	7.4202(6)	12.9674(11)	11.0930(7)	7.9085(4)
b (Å)	7.8105(6)	8.2138(7)	10.8351(7)	15.5190(7)
c (Å)	26.369(2)	14.9276(12)	26.8924(16)	9.2443(4)
β (°)	93.132(2)	106.429(2)	98.765(1)	114.271(1)
V ($Å^3$)	1525.9(2)	1525.0(2)	3194.6(3)	1034.29(8)
Z	4	4	8	4
μ (mm^{-1})	0.095	2.709	0.261	0.345
d_{calc} (g cm^{-1})	1.286	1.551	1.371	1.347
F(000)	632	728	1392	448
No. of measured refls.	12067	27516	22187	13144
No. of independent refls. (R_{int})	3301 (0.0265)	13586 (0.044)	5122 (0.025)	3014 (0.024)
No. of observed rfls. [I> 2σ(I)]	2892	9578	4656	2721
No. of parameters	206	379	215	134
Goodness-of-fit	1.03	0.99	1.08	1.03
$R_1[I > 2\sigma(I)]$	0.038	0.045	0.036	0.035
wR_2[all data]	0.099	0.105	0.091	0.085
$\Delta\rho_{max}$, $\Delta\rho_{min}$ ($eÅ^{-3}$)	0.71, -0.21	1.49, -0.86	0.45, -0.35	0.39, -0.21

Avance III 500 ^{1}H NMR **4e+5e** in DMSO-d_6

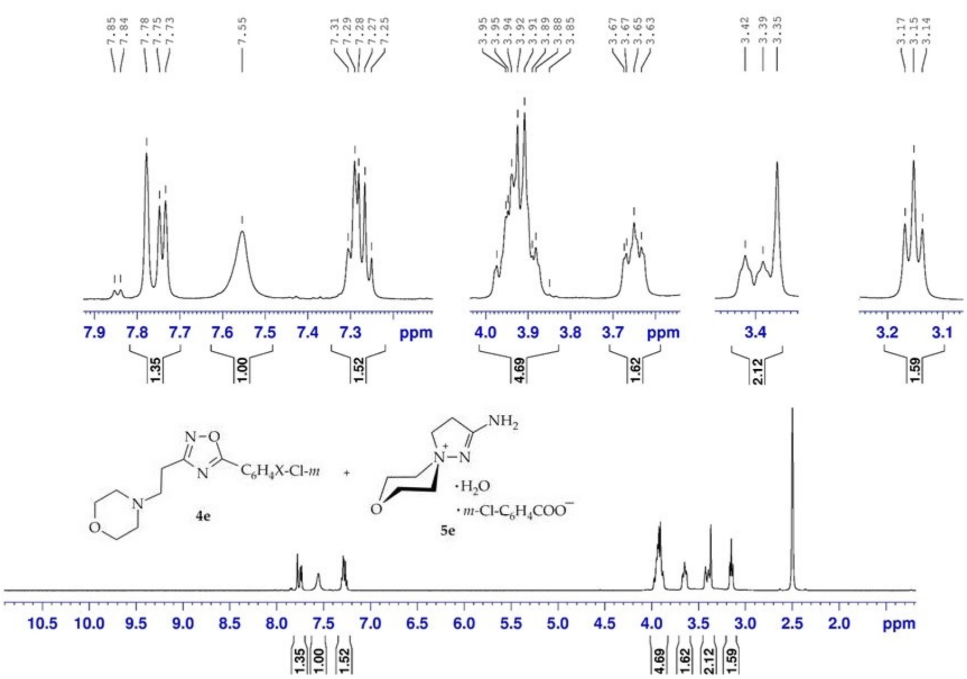

Avance III 500 ^{13}C NMR **4e** + **5e** in DMSO-d_6

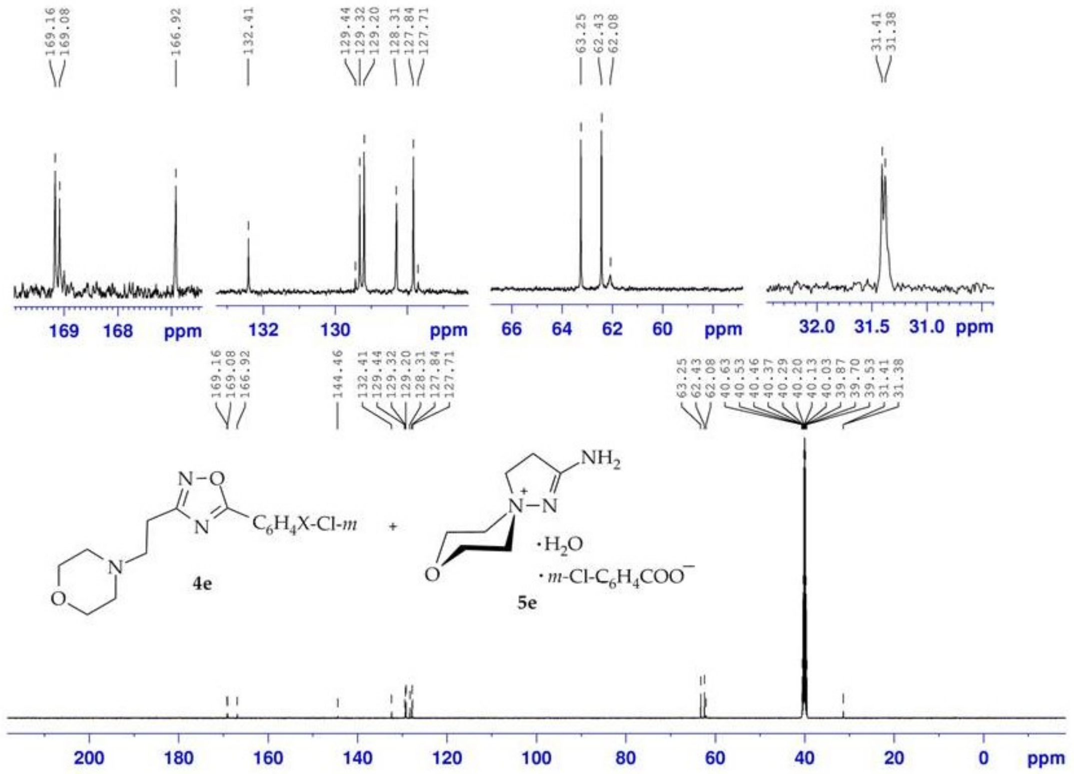

Avance III 500 ^{1}H NMR **5e** in DMSO-d_6

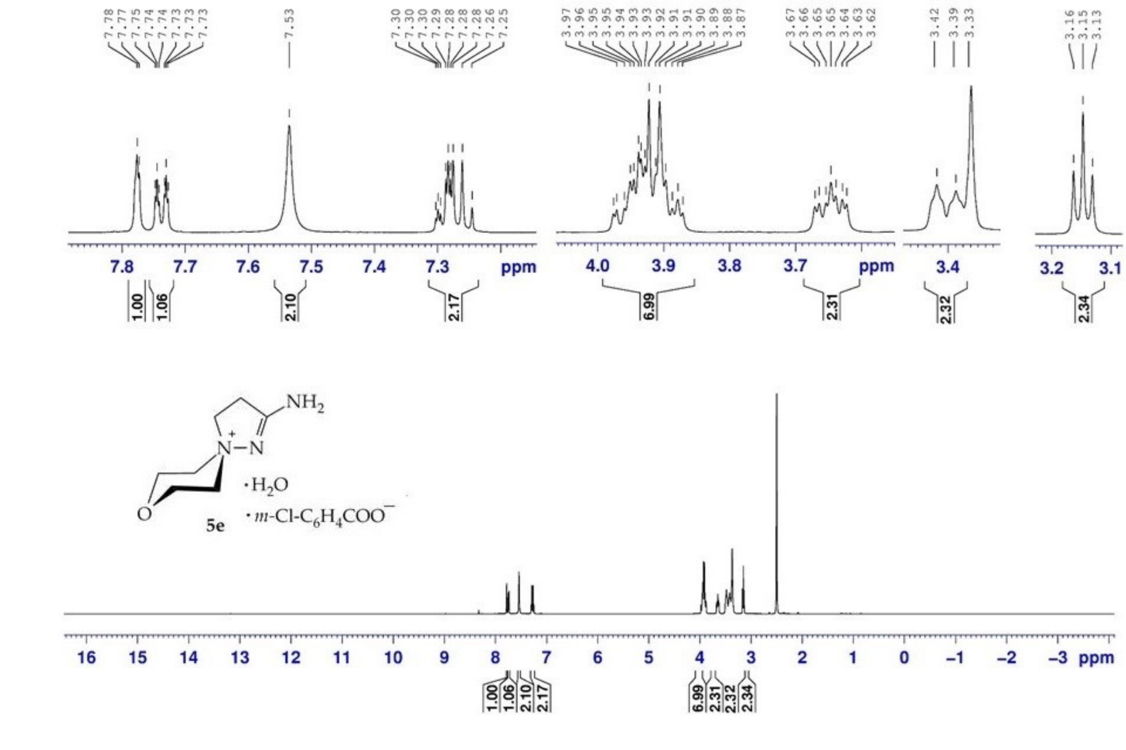

Avance III 500 ^{13}C NMR **5e** in DMSO-d_6

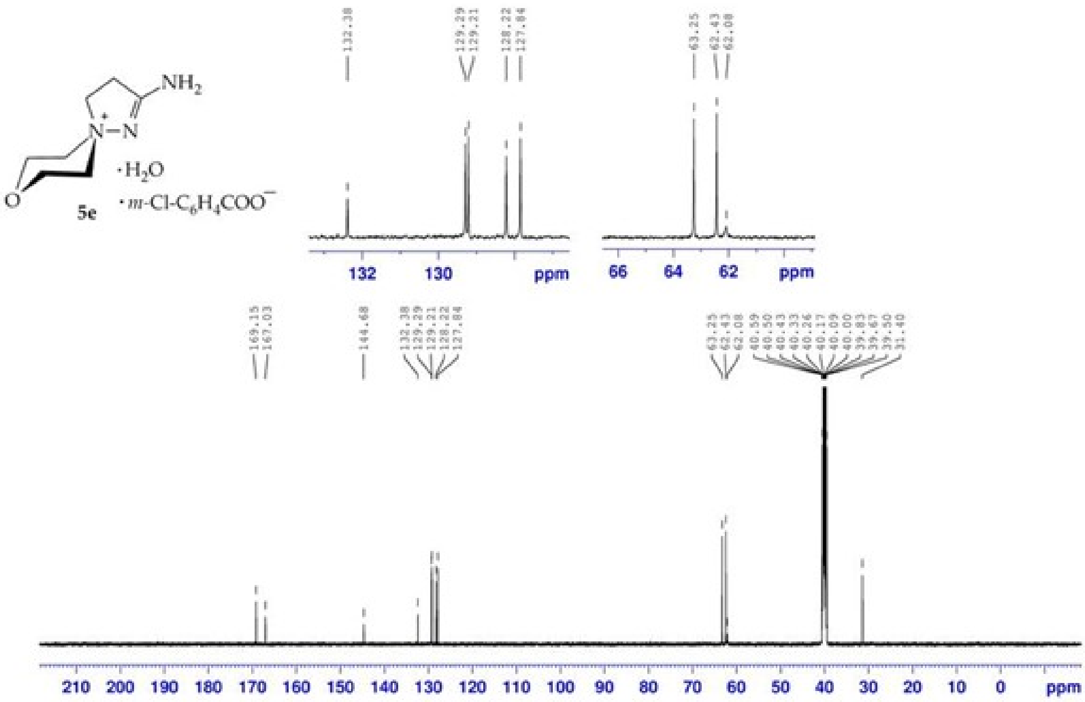

Avance III 500 ^{1}H NMR **6** in DMSO-d_6

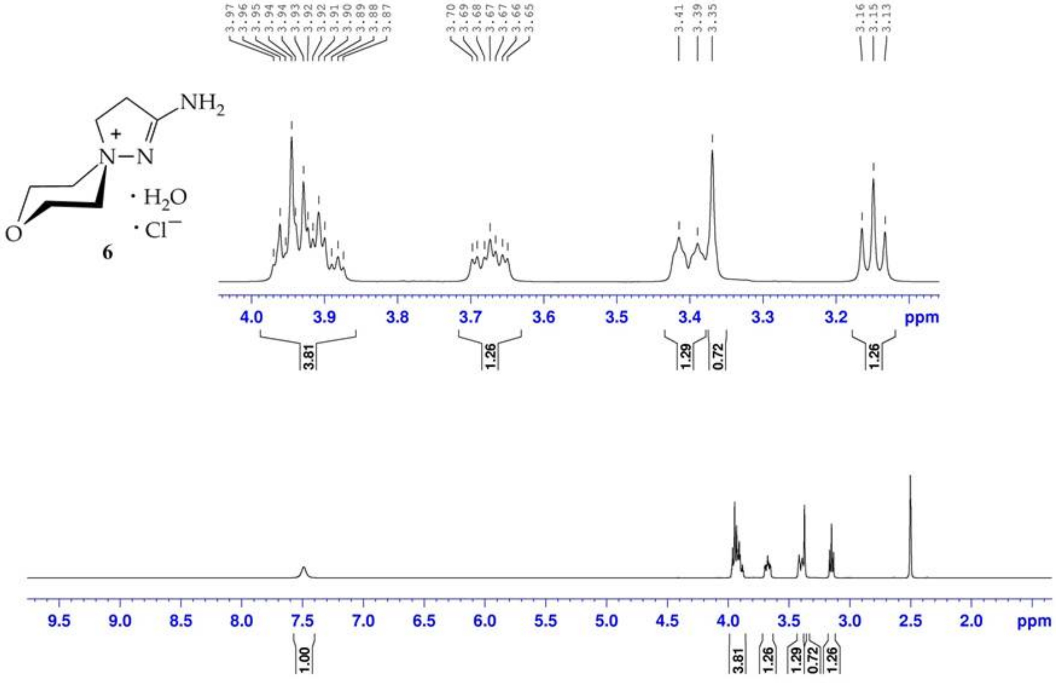

Avance III 500 ^{13}C NMR **6** in DMSO-d_6

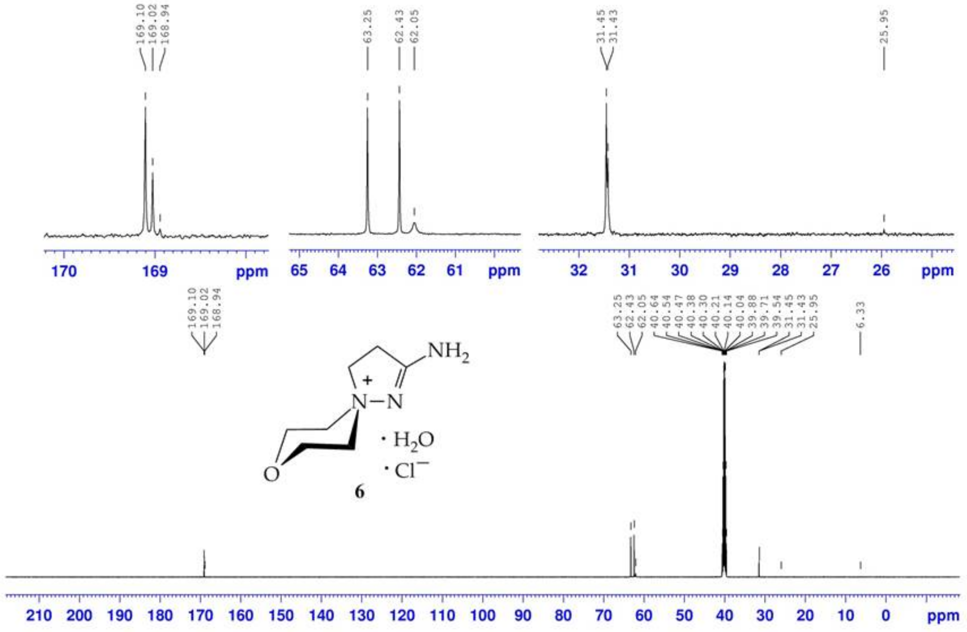

Supplement: Supplementary file 1 [file molecules-26-00967-s001.pdf]
